# Effects of Digestion, Cell Culture Media, and Mucous on the Physical Properties, Cellular Effects, and Translocation of Polystyrene and Polymethacrylate Nanoparticles

**DOI:** 10.3390/toxics11080708

**Published:** 2023-08-17

**Authors:** Zainab Jabor, Steven C. Sutton

**Affiliations:** 1 School of Pharmacy, Westbrook College of Health Professions, University of New England, 716 Stevens Ave, Portland, ME 04103, USA; 2 Department of Pharmaceutical and Administrative Sciences, School of Pharmacy, Westbrook College of Health Professions, University of New England, 716 Stevens Ave, Portland, ME 04103, USA

**Keywords:** nanoplastic, digestion, agglomeration, zeta potential, polystyrene, polymethacrylate, Caco-2, HT29-MTX-E12

## Abstract

The discovery of plastic and metal nanoparticles in organisms, foods, and beverages has generated numerous studies on the effects of these particles on the barrier cells and their subsequent absorption into the body. Following ingestion, nanoparticles travel down the gastrointestinal tract (GIT), and their physicochemical characteristics change in response to the change in proteins and pH during their digestion. We measured the translocation of digested nanoparticles across a co-culture monolayer of Caco-2 and various combinations (1:9, 5:5, and 9:1) of HT29-MTX-E12. The in vitro model of the intestine was used to determine the translocation of digested 20 nm polymethacrylate (PMA) particles and the accompanying monolayer barrier effects after a 72 h exposure. The in vitro digestion increased the agglomeration and hydrodynamic diameters and decreased the surface charge of the nanoparticles. For NH_2_-functionalized polymethacrylate nanoparticles (PMA-NH_2_), the diameters increased from 57 nm (water) to 3800 nm (media), or 2660 nm (chyme). These nanoparticles compromised the integrity of the monolayer (trans-epithelial electrical resistance, Lucifer yellow translocation) and translocated across all the cell ratio configurations. Digestion can have a large effect on nanoparticle agglomeration and surface charge. Excess mucous was not seen as a barrier to the translocation of PMA-NH_2_.

## 1. Introduction

The development of nanotechnology has enhanced the quality of some medical applications [1,2] and manufactured products that are common in daily use by consumers.

Plastic has invaded our culture as primary packaging, and, following weathering to nanoparticles, has been found in our food [3,4]. This ubiquitous material has been the subject of numerous studies searching for whether it will result in toxicity [1,2,5]. While irregularly shaped particles have shown, in some models, to produce more toxicity than spherical ones [6], relatively few studies have used nanoplastic formed from natural sources because of the difficulty in isolating the material into a narrow diameter range [7]. Polystyrene (PS) and polymethacrylate (PMA) nanoparticle spheres are the most frequently studied materials, commercially available with a narrowly defined nominal diameter of 20–50 nm, with or without a fluorescent label, and with surfaces that are uniform (not functionalized), or functionalized with carboxylic or amine groups. Positively charged nanoplastic has been reported to cause more harm to cells than negatively charged nanoplastic [8].

The nanoparticles that come into contact with the cells are also modified, depending on their environment [9]. For example, nanoplastic has been shown to readily bind with proteins and lipids to form a coating or corona. After ingestion, these nanoparticles travel down the gastrointestinal tract (GIT), and their physicochemical characteristics change in response to the changing milieu and pH [10] before they encounter the mucous that protects the barrier epithelial cells of the GIT. Digestion is therefore a crucial first processing step in determining the toxicity of these nanoparticles. The proteins and digestion products adsorb onto the surface of these materials, creating a corona that is different from the one in cell media [11]. The corona can cause agglomeration—which greatly increases the hydrodynamic diameter—and it can cause changes in the overall surface charge of the agglomerate [12,13].

The next step in the absorption journey of agglomerated nanoparticles is their interaction with the mucous lining of the GIT. Mucous secreted by goblet cells can slow nanoparticle absorption [14]. This protective negatively charged layer is made up of transmembrane mucins and gel-forming mucins. Positively charged PS nanoparticles have almost three times the binding and less than half the transit time through porcine jejunal mucous as anionic-charged PS nanoparticles [15]. Mucous consistency, composition, or quantity is variable and can be affected by disease (e.g., ulcerative colitis) [16]. The mucous-secreting cells are not as restrictive as epithelial cells, as shown by their very low transepithelial electrical resistance (TEER) [17].

While many studies have examined the effects of nanoplastic on target cells in isolation (i.e., not in combination with mucous-secreting cells, viz., [18,19,20]), a more physiologic model includes a mucous-secreting cell (e.g., HT29-MTX-E12). These model goblet cells are frequently used in combination with a model absorptive cell (e.g., Caco-2) to form a coculture monolayer [21]. Efforts to even more closely mimic the absorptive and inflammatory properties of the intestine have led to models including M-cells [22], monocyte-like cells [23], dendritic cells [6], and pluripotent stem cells [24]. While a more complex model may be relevant in nanoparticle toxicology [25], the nanoparticles must first cross the barrier cells to reach the monocytes and dendritic cells before causing the release of pro-inflammatory cytokines [6]. Therefore, we propose to study the translocation of two types of nanoplastic across the barrier cell monolayer intestinal model, as a function of the ratio of mucous-secreting cells to absorptive cells.

Most experiments that focus on studying the impact of plastic on the barrier properties of the GIT have used particles that are larger than 100 nm in diameter [26]. However, the agglomeration from digestion greatly increases the hydrodynamic diameter of 100 nm PS to larger than 400 nm [27]. Digestion effects on the zeta potential (ζ) contribute to the agglomeration of the nanoparticles. When the value of ζ falls into the range of +30 mV to −30 mV, agglomeration becomes much more likely [28]. Agglomeration can be the consequence of proteins and lipids adsorbed on the surface of nanoparticles; these forces are much weaker than the ones found between aggregation clusters and can be broken by physical force [29]. Anticipating the digestion-induced agglomeration, smaller-sized nanoparticles were used in this study (25–50 nm) to determine the effects of digestion and mucous on their translocation across a cell coculture.

Most experiments that use 50 nm PS also limit the cellular exposure to 24 h or less. But the nanoparticles that bind to membrane-bound mucous will not likely be excreted in the feces until the cells are sloughed off—about every 3–4 days [30]. Furthermore, studies have shown that nanoplastic is light, having a density close to that of the media, and may take several days for a significant number of particles to reach the cells in vitro [31,32]. This study examined the effects on cells after a 72 h incubation with the nanoparticles.

The studies reported here are attempts to fill some gaps in the reported literature by first digesting the nanoparticles, then using cocultures with different ratios of mucous-secreting cells to absorptive cells, and then subjecting them to an extended exposure of very small nanoplastic (25–50 nm).

## 2. Materials and Methods

### 2.1. Nanoparticle Properties

Polystyrene (PS) functionalized with surface carboxylic groups (PS-COOH, CA040NM, 43 ± 9 nm) and amine groups (PS-NH_2_, AM050NM, 53 ± 15 nm) were purchased from Magsphere (Pasadena, CA, USA) as aqueous suspensions of 10% solids. The fluorescently labeled polymethacrylate nanoparticles (PMA-COOH (30-02-252, 35 ± 6 nm) and PMA-NH_2_ (30-01-251, 39 ± 9 nm)) were purchased as 10 mg/mL aqueous suspensions from Micromod (Rostock, Germany). According to the manufacturer, the PMA-NH_2_ had amine groups on only 2.5% of the surface (most of the surface had exposed SO_3_H groups), resulting in a ζ of −23.8 at pH 7 (Figure S1). Note that the zeta charge reflects the net charge across the entire particle’s surface. We confirmed that the fluorescence did not leach from the nanoparticles during the experiments.

The hydrodynamic diameters and zeta potentials of the particles were determined in water, cell culture media (CCM, see below) and chyme by dynamic laser scattering with a 900 offset laser (NANO-flex II, W3504, Particle Metrix GmbH, Ammersee, Germany). The zeta potential or surface charge of nanoparticles was measured from pH 2 to 7 in water, CCM, and chyme using a Stabino Zeta (Microtrac GmbH, A0192, Meerbusch, Germany). According to the manufacturer, this measurement uses a streaming potential method: “Charged particles that generate an ion shell in polar liquids balance the charge between the particle surface and the liquid. The Stabino generates a charge separation by a liquid flow due to the oscillating motion of the plunger. The particles are immobilized on the walls of the PTFE chamber, and the liquid flow causes charge separation. As the mobile cloud of the double layer of the immobilized particles is pushed up and down, that oscillating ion cloud produces an alternating voltage at the two electrodes—the streaming potential—which is proportional to the zeta potential of the particles” [33]. The isoelectric points (pI) for each nanoparticle and media were determined from the pH-mV plots.

### 2.2. In Vitro Digestion Procedure

All the nanoparticles were subjected to saliva, gastric, and duodenal digestion according to Versantvoort [34]. All the chemicals and enzymes were purchased from Sigma. Briefly, the saliva, gastric and intestinal juices were made according to Supplemental Table S1. The nanoparticles were incubated in Falcon 15 mL tubes while tumbling head over heels at 37 °C. The digestion process started with combining the nanoparticles with 1 mL of prewarmed saliva juice for 5 min of tumbling at 37 °C. Then, 2 mL of prewarmed stomach juice was added, and the mixture was incubated for 30 min. Finally, 1 mL of prewarmed bile juice, 2 mL of prewarmed duodenum juice, and 28 mg of NaHCO_3_ were combined, then added to the saliva/gastric digestion and incubated for 3 h. The final product of the digestion (chyme) was diluted 1:9 with CCM to produce a chyme concentration at a non-toxic level [35]. While a 24 h incubation of a 9:1 Caco-2:HT29 coculture monolayer with undiluted chyme resulted in the maximum amount of LDH released, incubation with a 1:9 chyme:CCM dilution resulted in an LDH release that was similar to that of the spontaneous LDH release of untreated cells (see Supplemental Materials).

### 2.3. Cell Culture

Caco-2 (HTB-37, ATCC) and HT29 (HT29-MTX-E12, 12040401, ECACC) cells were seeded at 3 × 10^5^ cells per T75 flask, incubated humidified at 37 °C, 5% CO_2_, and refreshed three times weekly. The CCM consisted of DMEM (high-glucose GlutaMAX Supplement, Gibco 10566016, Life Technologies, Thermo Fisher Scientific, Grand Island, NY, USA), 10% fetal bovine serum (R&D Systems S1150H, Mineapolis, MN, USA), 1% penicillin-streptomycin (Gibco 15140122), and 1% nonessential amino acids (Gibco 1140050). The cells were examined under the microscope to track growth, and, once the cells reached at least 80% convergence, their viability and count were determined using a Guava 8HT flow-cytometer Luminex Corp, Seattle, WA, USA. Briefly, cells were detached from their growing flasks (T75, 5665-8175, USA Scientific, Ocala, FL, USA) after they were rinsed twice with 5 mL phosphate buffered saline (PBS) and then incubated with 2 mL trypsin/0.25% EDTA (25200056, Gibco) at 37 °C for 5 min. The trypsin was deactivated by adding 2 mL CCM to the detached cells. The cells were then centrifuged (Allegra X-22R, Beckman, Brea, CA, USA) at 500 relative centrifugal force for 5 min. The pellet was resuspended and counted using Guava^®^ ViaCount™ Reagent (NC1716261, Thermo Fisher Scientific).

Inserts (Falcon 08-0771-8, PET, 1 μm pore) were then seeded (1.8 × 10^5^ total cells/cm^2^) into cocultures of different ratios of Caco-2 to HT29 and placed in 12-well companion plates (Falcon 35303, Corning Life Sciences, Glendale, AZ, USA). The seeding ratios were (Caco-2:HT29): 9:1, 5:5, and 1:9 to examine a range of mucous-producing cells and secreted mucin. The CCM in the insert apical (AP) compartment (0.5 mL) and the well basolateral (BL) compartment (1.5 mL) were refreshed three times a week. After a 21-day incubation period, the cells had differentiated into mature epithelia and mucous-secreting cells to form a monolayer barrier on the insert membrane. The integrity of this monolayer was determined by measuring the transepithelial electrical resistance (TEER) using an EVOM2 (World Precision Instruments, Sarasota, FL, USA) and Lucifer yellow paracellular translocation across the monolayer (LY, CH lithium salt, Invitrogen, Life Technologies, Thermo Fisher Scientific L453, Carlsbad, CA, USA).

### 2.4. Nanoparticle Translocation Experiments

The experiments consisted of two studies separated by a few weeks: the first study comprised of PS nanoparticles, while the second study was made up of PMA nanoparticles. As shown in Figure S2, the experiments consisted of a 72 h incubation of cell monolayers with CCM containing the digested plastic nanoparticles. On day 21, the cocultures in the inserts were exposed to either (Study1): 0.5 mL of 143 µg/mL PS; or (Study2): 0.5 mL of 143 µg/mL PMA. The 12-well plates were then placed into a shaker (100 rpm/3 mm orbit, VWR 12620-926) inside an incubator (humidified, 37 °C, 5% CO_2_). At the end of the 72 h, basolateral and apical samples were collected and stored in microcentrifuge tubes at −80 °C until assayed. The cocultures were refreshed with cell culture media, returned to the incubator for one hour, and then the post-experiment TEER (post-TEER) was measured. Immediately following the TEER measurements, CCM was replaced in both the apical and basolateral compartments, with warmed Hank’s Balanced Salt Solution (HBSS). The apical HBSS contained 100 µM LY, and the plates were incubated for another hour. Basolateral samples were then collected, and the paracellular translocation of the LY, as well as the translocation of the PMA nanoparticles, were separately determined using a microplate fluorometer (Fluroskan Ascent 2.6, Thermo Fisher Scientific). The amount of LY (428/536 nm) or PMA (552/580 nm) translocated was determined using a standard curve made of LY- and PMA-dosing solutions and expressed as a percent of the dose.

### 2.5. Statistics

The data were analyzed for statistical significance using GraphPad Prism9 (GraphStats Technologies, Karnataka, India). The ANOVA results were compared using Tukey’s multiple comparison test and were considered significant at the *p* < 0.05 level. The pH titration curves of the surface charge were compared using the Kolmogorov–Smirnov test and considered significant at the *p* < 0.05 level.

## 3. Results

### 3.1. Effect of Digestion on Nanoparticle Hydrodynamic Diameter and Zeta Potential

The physical characteristics of the nanoparticles are summarized in Tables S2 and S3. While the physical size of these nanoparticles had been previously confirmed using SEM or TEM by us (Figure S3) or others [14,35,36], the more relevant dimension is the hydrodynamic diameter of the nanoparticles in the media in which the cell monolayer is incubated. The hydrodynamic diameter is the apparent diameter in media, which reflects any agglomeration and, together with the polydispersity index (PDI), indicates the degree of polydispersity of the particles. The nanoparticle hydrodynamic diameters were measured in water, CCM, and chyme. In general, the hydrodynamic diameters were larger in the CCM and chyme than in the water, presumably due to the adsorption of proteins and lipids from the CCM and digestion onto the nanoparticle surfaces [37]. In water, the hydrodynamic diameters of PS-COOH (48 nm, PDI 0.062) and PS-NH_2_ (56 nm, PDI 0.086) were not different from one another. A PDI theoretically can vary from 0–1, but a value larger than 0.3 reflects a sample that is not monodispersed. A sample can be polydispersed due to a varying degree of agglomeration. In some instances (e.g., PMA-COOH in chyme), the dispersion was too large to accurately produce a meaningful PDI value. For these instances, the maximum possible value of “1” was recorded. The extent of the polydispersity was reflected in the intensity vs. diameter sample plot for PS-uniform in CCM (see Figure S4).

**Figure 1:**
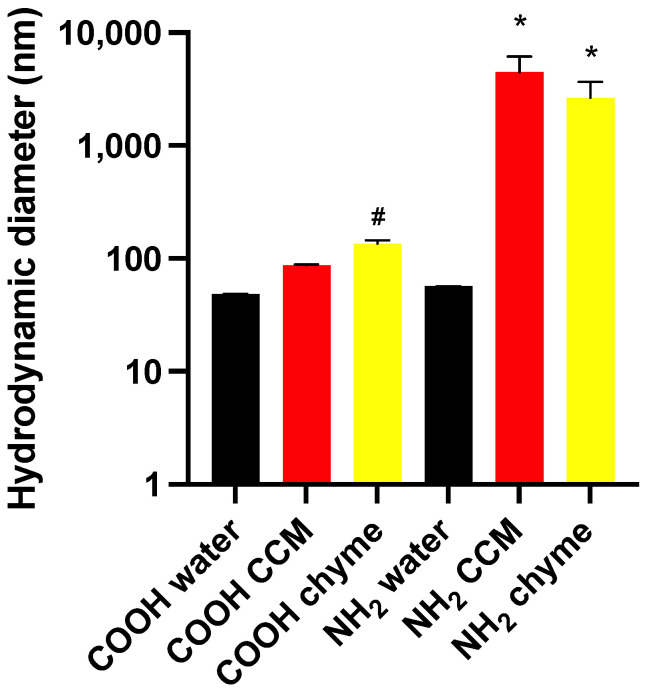
Hydrodynamic diameters of PS nanoparticles in water, CCM, and chyme. Mean ± SD measurements on at least three separate samples, * different from water, # different from NH_2_ (chyme).

In the CCM and chyme, the hydrodynamic diameters of PS-NH_2_ were 4440 nm (PDI 0.071) and 2060 nm (PDI 0.161), respectively, which were larger than in the water (*p* < 0.05). The hydrodynamic diameters of PS-COOH in CCM (87.7 nm, PDI 0.128), or in chyme (135 nm, PDI 0.649), were not different from those in water, although the polydispersity was more evident in the chyme samples. The diameter of PS-NH_2_ in the chyme was larger than that of PS-COOH in the chyme (*p* < 0.05). Figure 1 summarizes these findings, and Table S2 includes the PDI values.

**Figure 2:**
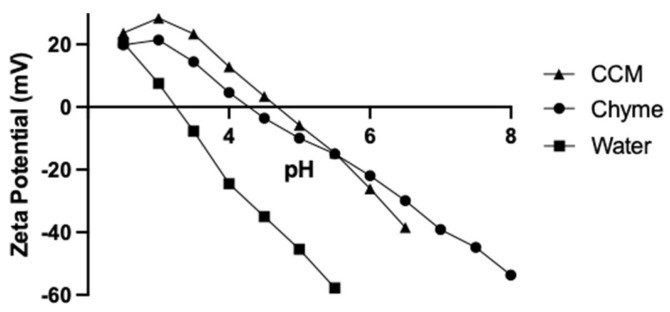
pH-titration curves of PS-COOH as measured in water, CCM, and chyme.

The zeta potential (ζ) of nanoparticles was also measured in water, CCM, and chyme. Since the charge changed with the pH of the media, titrations were completed over the pH range of 2 to 7. The isoelectric point (pI) is the pH on the titration curve where the nanoparticles have no net charge. The negatively charged groups on the nanoparticle surface are protonated at low pH and ionized at high pH. As shown in Figure S5, the ζ at a given pH also depends on the number of nanoparticles titrated. The larger the concentration of nanoparticles in the preparation, the smaller the fraction of the groups that become protonated at a given pH. The pI is relatively insensitive to the nanoparticle concentration. When the value of ζ falls into the range of 30 mV to −30 mV, agglomeration becomes much more likely [28]. Agglomeration can be the consequence of proteins and lipids adsorbed on the surface of nanoparticles; these forces are much weaker than the ones found between aggregation clusters and can be broken by physical force [29]. The digested corona can reduce the surface charge on these particles. While digestion had little effect on the ζ of most nanoparticles (e.g., chyme vs. water), digestion either increased (negatively charged particles) or decreased (positively charged particles) the pI (Table S3). One possible explanation is that digestion resulted in the adsorption of small protein fragments which covered a significant fraction of the charged groups, resulting in a pI achieved with fewer hydronium ions, as shown for PS-COOH (Figure 2). Due to the digestion of proteins, for nearly all the nanoparticles, the values of pH6 ζ became less negative in CCM and chyme (Table S3). The values of pI in the water were increased when measured in the chyme for all the nanoparticles except PS-NH_2_. The PMA-NH_2_ pH6 ζ in the water became less negative in the chyme (*p* < 0.05), and even less negative in the CCM (*p* < 0.05, Figure S6). This was also the case for the PMA-COOH (Figure S7).

**Figure 3:**
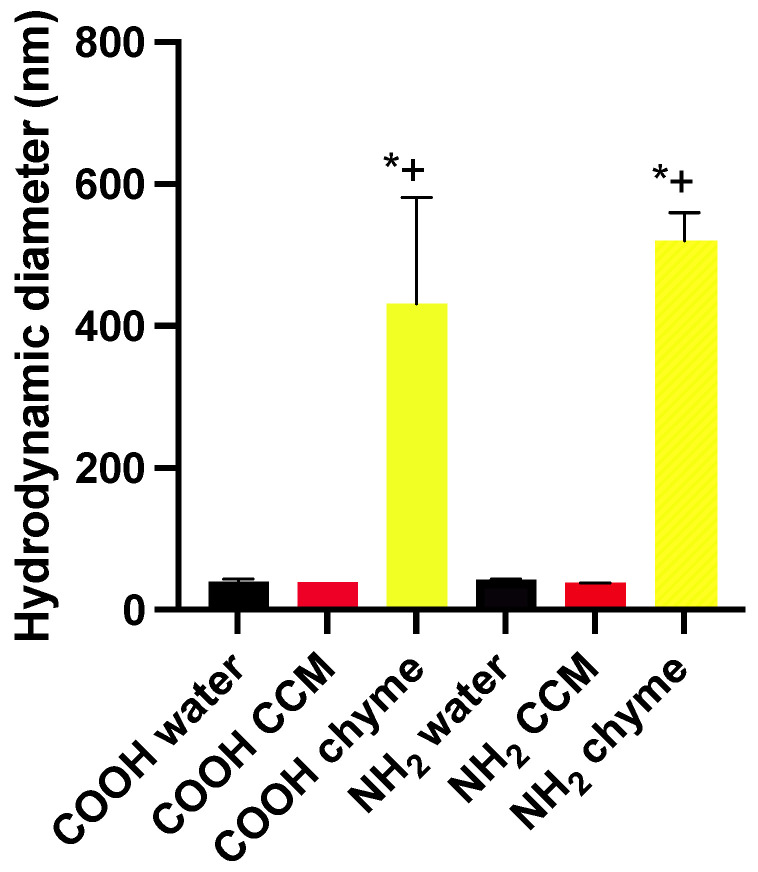
Hydrodynamic diameters of PMA nanoparticles in water, CCM, and chyme. Mean ± SD measurements on at least three separate samples, * different than water, + different than CCM.

The hydrodynamic diameters of the PMA-COOH in water (40 nm, PDI 0.794) and the PMA-NH_2_ (42 nm, PDI 0.053)) were not different from each other. The hydrodynamic diameters in the CCM of the PMA-COOH (39 nm, PDI 0.171) and the PMA-NH_2_ (37 nm, PDI 0.436) were not different from each other. And the hydrodynamic diameters in the chyme of the PMA-COOH (432 nm, PDI 1) and the PMA-NH_2_ (520 nm, PDI 0.789) were not different from each other, but both were significantly larger than their respective nanoparticles in the CCM (*p* < 0.05) (Table S2, Figure 3).

### 3.2. The Effects of Nanoparticle Exposure on the Monolayer Barrier Properties and Translocation

**Figure 4:**
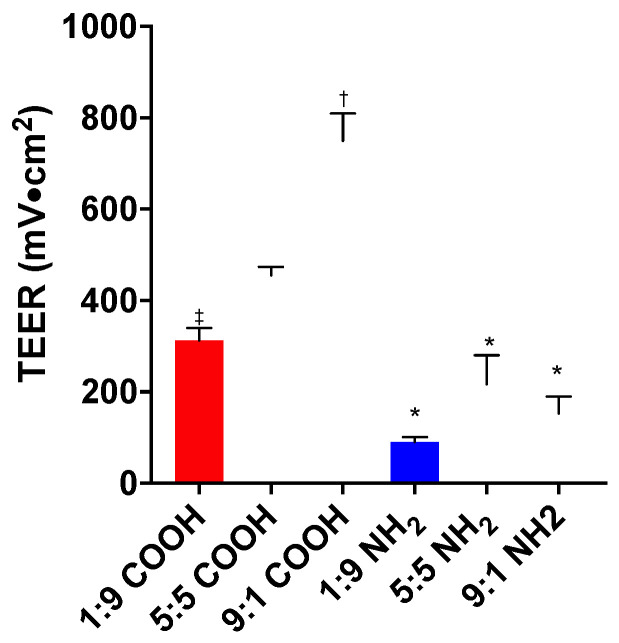
Post-experiment TEER after 72 h exposure to 50 nm PS in monolayers of different Caco-2:HT29 cell ratios. Mean ± SD of at least three unique inserts, * different than COOH (with the same cell ratio), †,‡ different than 1:9 cell ratio (with the same nanoparticles).

The pre-experiment TEER (pre-TEER) ranged from >200 for the Caco-2:HT29 ratio 1:9 to >600 Ω·cm^2^ for the 9:1 ratio (Table S4). The pre-TEER for the 9:1 monolayer was significantly larger than the 5:5 or 1:9 monolayer (*p* < 0.05). This reflected the much tighter monolayer of Caco-2 cells than the goblet HT29 cells, as the cell–cell connectivity of Caco-2 cells is greater than for HT29 cells [21,38], and confirmed the different cell ratios employed in this study. Compared to the pre-TEER, PS-NH_2_ caused a significant decrease in the post-experiment TEER (post-TEER) at all the cell ratios (*p* < 0.05). The post-TEER after exposure to PS-NH_2_ was also lower than the post-TEER after exposure to PS-COOH (*p* < 0.05). PS-COOH did not cause a decrease in TEER (Figure 4). No TEER results are shown for the PMA study, due to the malfunctioning of the EVOM instrument.

**Figure 5:**
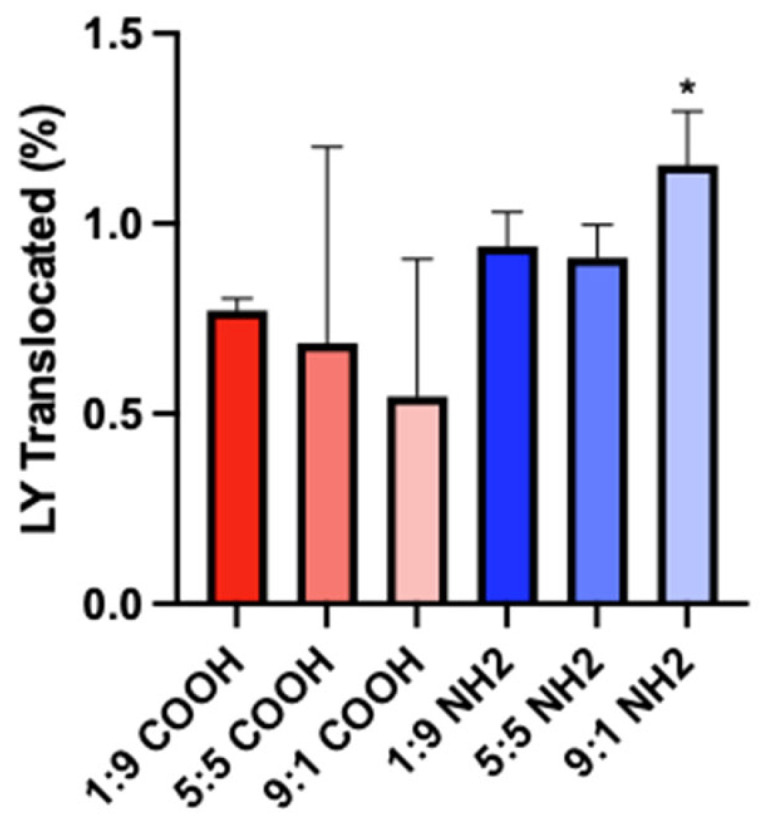
Post-experiment LY translocation after a 72 h exposure to PMA-COOH and PMA-NH_2_ in monolayers of different Caco-2:HT29 cell ratios. The amount translocated into the BL compartment relative to the dose administered to the AP compartment is shown as a percent (%). Mean ± SD of at least three unique inserts, * different from PMA-COOH.

The pre-experiment translocation of Lucifer yellow (pre-LY) was <1% for all the mucous conditions, consistent with an intact barrier (e.g., Table S5). Compared to the PMA-COOH, the post-experiment LY (post-LY) translocation was significantly greater for the PMA-NH_2_ in the 9:1 cell ratio but not in the 1:9 or 5:5 cell ratio conditions (*p* < 0.05) (Table S5, Figure 5).

**Figure 6:**
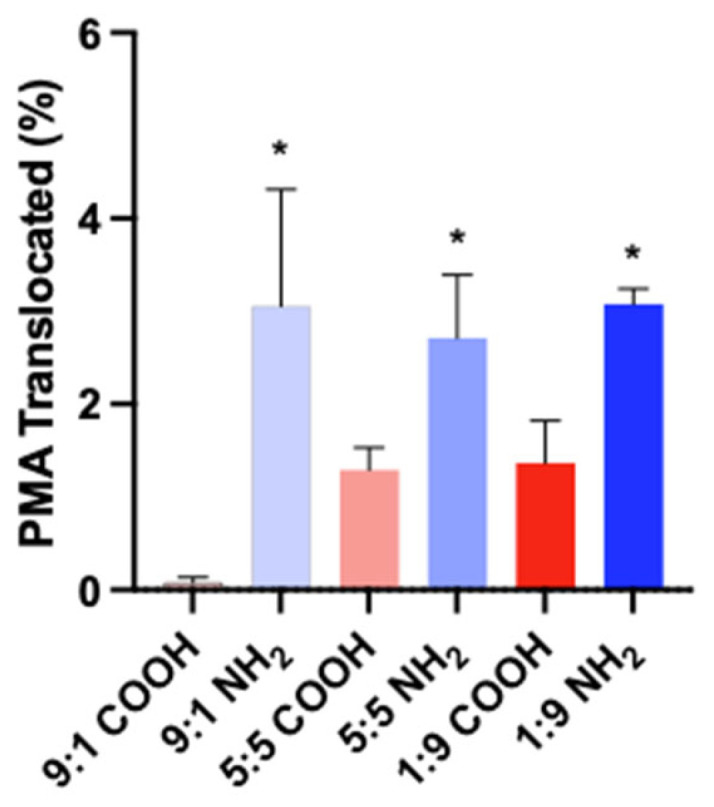
Post-experiment PMA translocation after a 72 h exposure to PMA-COOH or PMA-NH_2_ through monolayers of different Caco-2:HT29 cell ratios. The amount of nanoparticles translocated into the BL compartment is expressed as a percent (%) of the amount administered to the AP compartment. Mean ± SD of at least three unique inserts, * different from PMA-COOH at the same cell ratio.

The PMA nanoparticles that translocated across the monolayer and into the basolateral compartment were quantitated by fluorescence and expressed as a percent of the administered dose in the apical compartment. Very little PMA-COOH translocated in any of the cell ratio configurations (0.08 to 1.4%). Significantly more PMA-NH_2_ translocated in every cell ratio configuration (2.7 to 3.1%, *p* < 0.05). The percent of PMA-NH_2_ translocated was not different among the cell ratio configurations (Figure 6, Table S6).

## 4. Discussion

This study examined the effects of a 72 h exposure of digested plastic nanoparticles on cocultures of differing ratios of the intestinal epithelial cell, Caco-2, and the mucous-producing goblet cell, HT29. Mucous serves as a protective barrier that some researchers have shown decreases the diffusion of plastic nanoparticles to the cell surface [14]. Liu et al. showed the impact of in vitro digestion on the agglomeration and surface charge of plastic nanoparticles [27]. Liu et al. also showed that the toxicity of 100 nm PS on Caco-2 monocultured monolayers was decreased after the nanoparticles were digested. In contrast, Walczak found that digestion increased the translocation of 50 nm PS nanoparticles across a Caco-2 and HT29 co-culture (3:1) monolayer [35]. Their explanation for the increased translocation was that, compared to the corona that the nanoparticles developed in CCM, the digestion reduced the corona and the size of proteins adsorbed onto the nanoparticles. They did not report the effect of digestion on the agglomerate mean diameter or charge. In a previous study, we detected a 10–12% translocation of undigested fluorescent 50 nm PS-uniform nanoparticles across a monolayer consisting of Caco-2 cells in 24 h [39]. The inserts alone did not impede the diffusion of pristine (not digested) PMA from the AP compartment to the BL compartment: 54% of the AP dose was accounted for in the BL compartment after a 24 h incubation (see Supplemental Information). However, digestion decreased this diffusion, resulting in only 28% of the dose diffusing into the BL compartment over this same time. This may be due to the agglomeration during digestion resulting in a more buoyant particle that takes much longer to gravitate to the filter, and/or the slower translocation of larger agglomerate particles through the filter’s pores. Yet, in this study, we observed only about 1–3% translocation of PMA nanoparticles over 72 h (Figure 6). In comparing these studies, we must factor (1) the effect of mucous-secreting cells in a coculture with the resulting amount of mucous secreted, and (2) the effects of digestion on the formation of agglomerate size and charge. In this study, the digested PMA nanoparticles resulted in larger mean agglomerate diameters in chyme than in CCM. However, since the chyme PDI were large, there was a large degree of polydispersity (Table S2). The charge was also affected by digestion: compared to the pI from titration in CCM, the pI from titrations in chyme were reduced for PS-NH_2_, PS-COOH, and PMA-COOH (Table S3). Regardless of the effects of digestion on surface charge or particle size, since all nanoparticles that are ingested will be subject to digestion, it seems reasonable to always include a digestion step before exposing cells to nanoparticles.

As expected, the pre-TEER value for the (Caco-2:HT29) 1:9 cell ratio was the lowest, followed by the 5:5 cell ratio, with the 9:1 cell ratio having the largest value of TEER (Table S4). Yet the pre-LY values were similar across all the cell ratios. LY has an mw of 457, 14 nitrogen and oxygen atoms, and two negative charges at cell culture pH. While these characteristics hinder its transcellular transport, it is the quintessential paracellular transport marker used in cell culture [40]. Since TEER measures only ion passage, it is possible that LY translocation could be low, even with low TEER. However, a disruption of the tight junction proteins could result in high LY permeability and low TEER. Evidently, the cell interactions for all the cell ratios were sufficient to exclude this paracellular transport marker.

The decrease in PS-NH_2_ TEER (post-experiment vs. pre-experiment, Table S4) has been reported by others for PS-NH_2_ [35]. Those authors and others [41,42] have attributed these changes in TEER and translocation to a disruption of the barrier function in the monolayer. The disruption is similar to that observed for permeation enhancers and appeared to affect the tight junctions between cells [43,44,45].

The PMA-NH_2_ were translocated across the monolayer to a greater extent (3-fold, *p* < 0.01) than the PMA-COOH and caused enough monolayer barrier disruption to result in an increased post-LY paracellular translocation, again relative to the PMA-COOH (Table S6). Very little PMA-COOH translocated under any mucous condition.

The mucous condition did not appear to make any difference in the PMA-NH_2_ translocation, and, despite the effect of digestion on reducing the surface charge, more PMA-NH_2_ translocated than did PMA-COOH. The finding that positively charged nanoparticles caused more cell toxicity than negatively charged nanoparticles is consistent with findings by others [8,36]. Still, it was interesting to note that even after digestion, and the associated increased diameter and diminished surface charge, the positively charged PMA-NH_2_ evidently reached the cells, causing a greater disruption of the monolayer barrier and more translocation than the PMA-COOH nanoparticles.

## 5. Conclusions

The literature explosion around the subject of nanoparticles in general, and specifically nanoplastics, makes a thorough understanding all but impossible. The concern over the global pollution of pervasive and ubiquitous plastic has justified the research to determine the toxicity of nanoparticles reported in whole animals. However, confusing and often contrary results have raised doubt about how the nanoparticles damage and cross the barrier cells in the intestine and affect the innate immune system [46].

The purpose of this study was to examine the translocation of nanoplastic across an in vitro model of the intestine. It is not a statement on the medical use of polymeric nanoparticles, as they represent a tiny fraction of the plastisphere [47]. The longer 72 h incubation reflected the in vivo sloughing of intestinal cells every three days [30], and the simulation that, for in vitro models, it takes three or more days for the low-density plastic nanoparticles to be deposited on the cells [31,32]. This was explained by the buoyancy of the nanoplastic agglomerates and the distance that the nanoparticles had to diffuse to reach the cells on the insert filter [48]. The use of an inverted cell model configuration somewhat addresses this problem [19]. It is not known to what extent buoyancy and diffusion are obstacles to the impact of orally ingested nanoplastic. A limitation of this work is the absence of confocal microscopy, which could support the findings of PMA-NH_2_ on the cell monolayer barrier.

This work has shown that the translocation of digested, amine-functionalized nanoplastic can reach, affect damage, and cross an in vitro intestine model, even with a 9-fold greater number of mucous-secreting cells than typically used [21]. The implication of positively charged nanoplastic crossing the intestinal barrier, even in extreme cases of excessive mucous production (viz. Crohn’s disease, ulcerative colitis, cancer), warrants further investigation.

## Data Availability

The data presented in this study are available on request from the corresponding author.

## Supplementary Materials

The following supporting information can be downloaded at: https://www.mdpi.com/article/10.3390/toxics11080708/s1. 1. LDH assay to determine the non-toxic level of diluted chyme. 2. Translocation of nonfunctionalized PMA nanoparticles through the insert filter. 3. Figure S1. ζ vs. pH for PMA-NH2, supplied by Micromod. 4. Table S1. Composition of in vitro fed digestion model (mg amounts based on 10 mL juice). 5. Figure S2. Timeline of the nanoparticle translocation experiments. 6. Table S2. Hydrodynamic diameters (nm) and polydispersity index (PDI) for nanoparticles determined in water, cell culture media (CCM), and digestion chyme. 7. Table S3. Zeta potential (ζ, mV) at pH 2.5 and 6 and isoelectric point (pI, pH) of nanoparticles determined in water, CCM, and chyme. The pI results are from pH titrations. 8. Figure S3. SEM micrographs of the PS-COOH nanoparticles. 9. Figure S4. Intensity vs. diameter plot for PS-uniform in CCM showing the polydispersity associated with a PDI of 1. 10. Figure S5. pH-titration curves for PS-uniform showing the effect of nanoparticle mass (50–500 µg) on the shape of the curve, all intersecting at the same isoelectric point, pI. 11. Figure S6. pH-titration curves of PMA-NH2 measured in water, CCM, and chyme. 12. Figure S7. pH-titration curves of PMA-COOH measured in water, CCM, and chyme. 13. Table S4. Transepithelial electrical resistance (TEER) measured before and after exposure to PS nanoparticles. 14. Table S5. Pre- and Post-experiment Lucifer yellow (LY) translocation shown as the percent (%) LY in the basolateral compartment in one hour, relative to the administered dose. 15. Table S6. Nanoparticle translocation shown as the percent (%) of the amount translocated into the basolateral compartment in one hour, relative to the administered dose to the apical compartment.
