# Supplementary material for: Effects of Digestion, Cell Culture Media, and Mucous on the Physical Properties, Cellular Effects, and Translocation of Polystyrene and Polymethacrylate Nanoparticles
Source: Toxics. 2023 Aug 17;11(8):708. doi: 10.3390/toxics11080708 (PMC10458608; doi:10.3390/toxics11080708)
Supplement: Supplementary file 1 [file toxics-11-00708-s001.zip › toxics-2442203-supplementary.pdf]

Supplemental Information.

1. LDH assay to determine the non-toxic level of diluted chyme.

Samples were assayed for LDH with the colorimetric CyQUANT LDH cytotoxicity assay kit (invitrogen C20300). Briefly, 50 µL samples were incubated in triplicate with 50 µL of the reaction mixture for 30 min in the dark, followed by 50 µL of stop solution before absorbance was measured at 490nm and 680nm. A positive control, a maximum LDH release sample, a CCM blank and an untreated sample (spontaneous LDH release) were included.

Exposure of cell monolayer to full-strength chyme and to diluted chyme (1:9 with CCM). Fully differentiated 9:1 Caco-2:HT-29 coculture monolayers on inserts were exposure to either full strength chyme, 9:1 diluted chyme or CCM, and incubated (humidified, 37°C, 5% CO_2) for 24h. Apical samples were then collected and LDH release determined.

LDH released from cells incubated with the diluted chyme was 1.37 ± 0.24 absorption units (ABS) compared to that of the spontaneous level of LDH release in untreated cells 1.49 ± 0.02 ABS. Whereas the samples incubated with full-strength chyme exhibited ABS levels that were similar to the maximum LDH release readings of >4 ABS.

2. Translocation of nonfunctionalized PMA nanoparticles through the insert filter.

The inserts employed in this study were used to determine the translocation of fluorescent non-functionalized PMA nanoparticles following a 24h incubation (humidified, 37°C, 5% CO_2), at the same concentration as the diluted chyme dosing solutions. The following conditions were examined with the filters only (no cell monolayer): pristine PMA, digested PMA diluted 9:1 with PBS, digested PMA diluted 9:1 with CCM. Following the 24h incubation, 100 µL samples of the basolateral (BL) compartment (total 1.5 mL) were collected and fluorescence measured (530/590) along with a standard curve and expressed as a percent of the dose.

Pristine PMA translocated across the filter and 54 ± 12% of the dose was found in the BL compartment. Far less digested PMA diluted 1:9 with CCM translocated across the filter: 28 ± 1.8%. This may be due to agglomeration during digestion resulting in a more buoyant particle that takes much longer to gravitate to the filter, and/or the slower translocation of larger particles through the filter's pores.

3. Figure S1. ζ vs pH for PMA-NH2, supplied by Micromod.

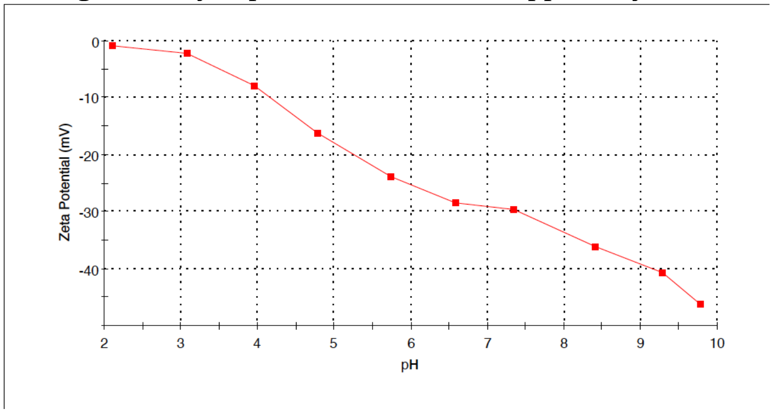

4. Table S1. Composition of in vitro fed digestion model (mg amounts based on 10mL juice).

**Chemical**	**Saliva**	**Gastric**	**Duodenum**	**Bile**
Amylase	2.90			
BSA		10.00	10.00	18.00
Bile				300.00
CaCl2		3.02	1.51	1.66
glucosamine		3.30		
glucose		6.50		
glucuronic acid		0.20		
KCl	8.96	8.24	5.64	3.76
KH2PO4			0.80	
KSCN	2.00			
lipase			15.00	
Mucin type2	0.25	30.00		
Na2SO4	5.70			
NaCl	2.98	27.52	70.12	52.59
NaH2PO4	8.88	2.66		
NaHCO3	16.94		33.88	58.02
NH4Cl		3.06		
pancreatin			90.00	
pepsin		25.00		
Urea	2.00	0.85	1.00	2.50
Uric acid	0.15			

5. Figure S2. Timeline of the nanoparticle translocation experiments.

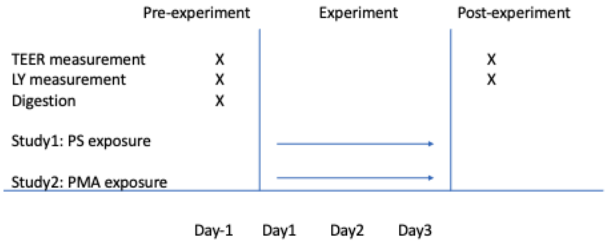

6. Table S2. Hydrodynamic diameters (nm) and poly dispersity index (PDI) for nanoparticles determined in water, cell culture media (CCM), and digestion chyme.[1]

Nanoparticle	Water	CCM	Chyme
PS-COOH	48 ± 0.231 (0.062)	87.7 ±0.872 (0.128)	135 ± 9.61 (0.649)
PS-NH_2	56.1 ±0.932 (0.086)	4440 ± 1530* (0.071)	2060 ±767*†‡ (0.161)
PMA-COOH	39.7 ± 3.01 (0.794)	39.1 ± 0.153 (0.171)	432 ± 150*† (1)
PMA-NH_2	42.4 ± 0.404 (0.053)	37.4 ± 0.4 (0.436)	520 ± 39*† (0.789)

[1] Mean ± SD of at least three measurements were made of each nanoparticle and media. * different from water ($p<0.05$), † different from CCM ($p<0.05$), ‡ different from PS-COOH (same media).

7. Table S3. Zeta Potential (ζ, mV) at pH 2.5 and 6 and Isoelectric Point (pI, pH) of nanoparticles determined in water, CCM and chyme. The pI results are from pH titrations.[1]

Nanoparticles	ζ at pH 2.5 (mV)			ζ at pH 6 (mV)			pI (pH)		
	Water	CCM	Chyme	Water	CCM	Chyme	Water	CCM	Chyme
PS-COOH	19 ± 1.4	25	21 ± 4.7	-48 ±17	-64	-26 ± 5.3	3.3	4.7*	4.3*
PS-NH_2	22	22	21 ± 2.6	-25	-25	-19 ± 1.2	2.1	4.2	4.1
PMA-COOH	-5 ± 0	10 ± 21	5	-87 ± 12	5*	-38*	2	6.3*	2.8*†
PMA-NH_2	14 ± 15	25	25 ± 3.8	-45 ± 34	-6*	-33* ± 3.3	3.5	5.3	3.7

[1] Where indicated, mean ± SD of three or more independent measurements are shown. The pH titration curves of surface charge were compared using the Kolmogorov-Smirnov test and considered significant at the $p<0.05$ level. * different than water ($p<0.05$), † different than CCM ($p<0.05$).

8. Figure S3. TEM micrographs of the PS-COOH nanoparticles[1].

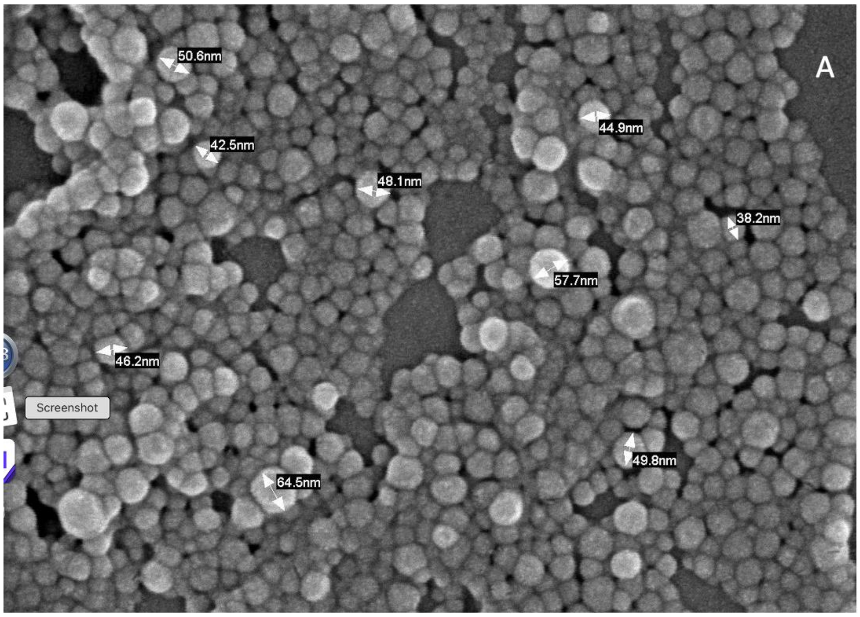

[1] Sample was applied to a 300 mesh copper grid with a carbon support film and negative stained with 2% uranyl acetate (JEOL JEM 1010, Peabody, MA, USA, operated at 80 kV).

9. Figure S4. Intensity vs diameter plot for PS-uniform in CCM showing the polydispersity associated with a PDI of 1.

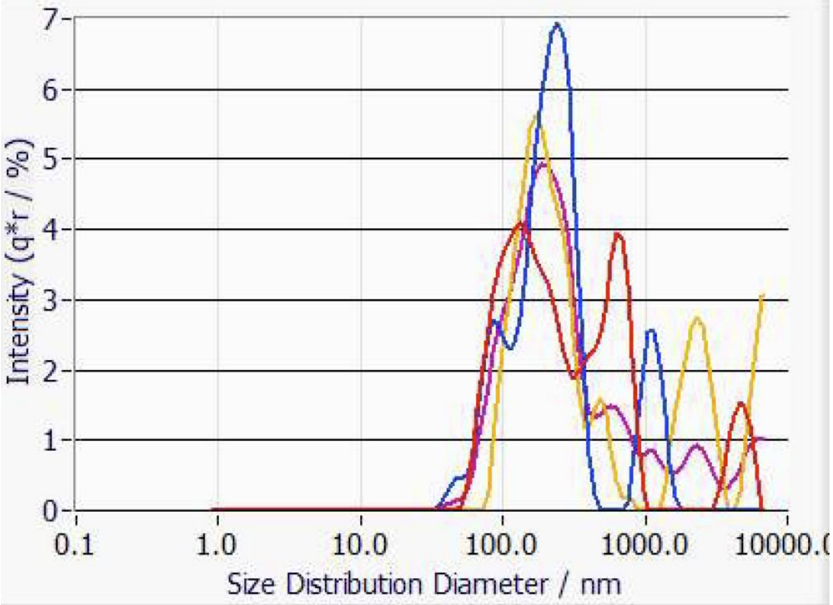

10. Figure S5. pH-titration curves for PS-uniform showing the effect of nanoparticle mass (50-500 μg) on the shape of the curve, all intersecting at the same isoelectric point pI.

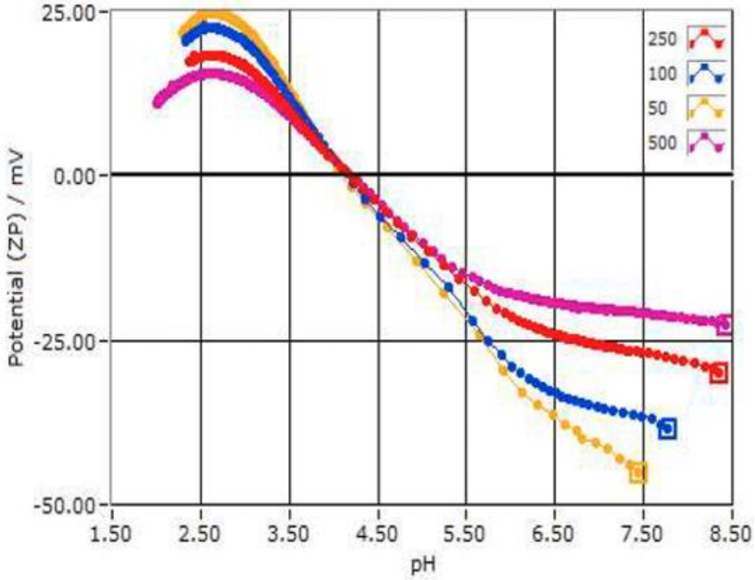

11. Figure S6. pH-titration curves of PMA-NH2 measured in water, CCM and chyme.

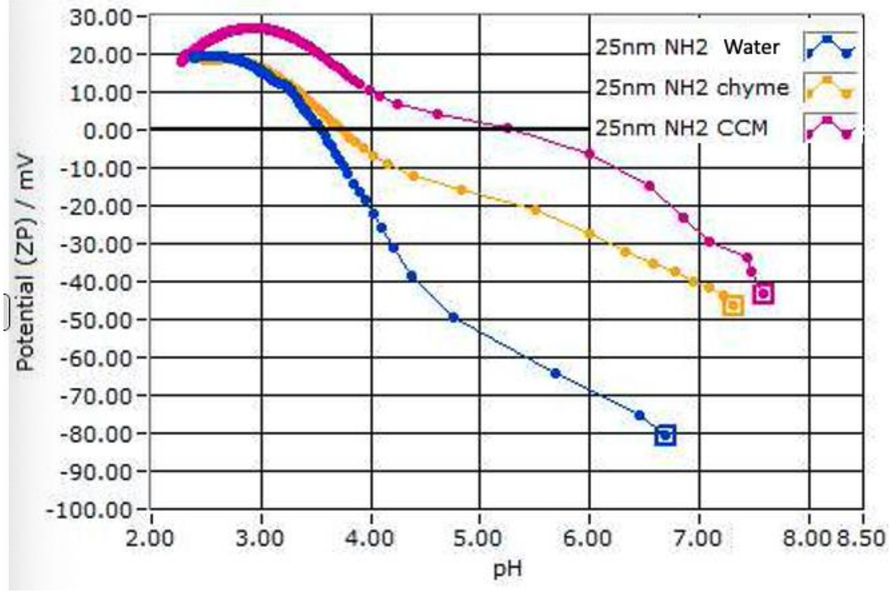

12. Figure S7. pH-titration curves of PMA-COOH measured in water, CCM and chyme.

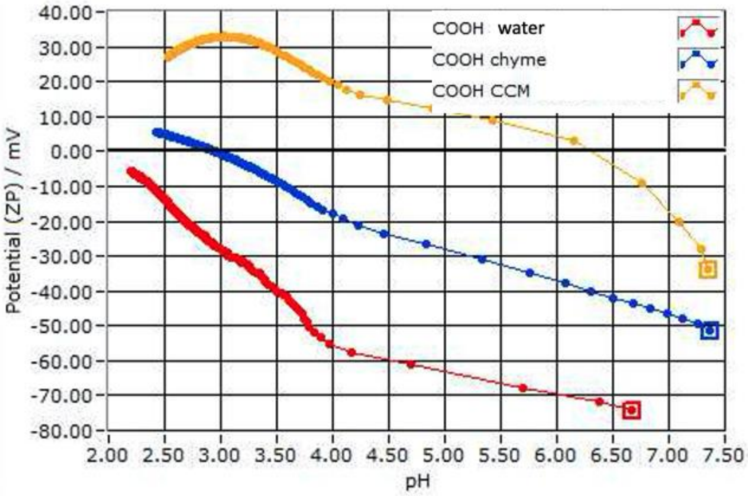

13. Table S4. Transepithelial electrical resistance (TEER) measured before and after exposure to PS nanoparticles.[1]

Seeding Ratio (Caco-2:HT29)	Pre-TEER (Ω•cm²)		Post-TEER (Ω•cm²)	
	PS-COOH	PS-NH_2	PS-COOH	PS-NH_2
1:9	239 ± 13.5 +	243 ±18.0 +	310 ± 27 ‡	90 ± 10.6 *#
5:5	315, 432 +	324, 450 †	441, 468	171, 261 *#
9:1	600± 44 †	723 ± 18.7 †	743 ± 59 †#	151 ± 37 *#

[1]Mean ± SD of at least three inserts, except for 5:5 seeding ratio.
Pre-TEER within nanoparticle type comparisons: †different than pre-TEER 5:5, p<0.05; +different than pre-TEER 9:1, p<0.05.
Post-TEER within nanoparticle type comparisons: †different than post-TEER 5:5, p<0.05; ‡different than post-TEER 9:1.
Post-TEER between nanoparticle type comparisons: *different than post-TEER PS-COOH, p<0.05.
Pre *vs* post TEER comparisons: #different than pre-TEER (same nanoparticle type, same cell seeding ratio), p<0.05.

14. Table S5. Pre- and Post-experiment Lucifer Yellow (LY) translocation shown as the percent (%) LY in the basolateral compartment in one hour, relative to the administered dose.[1]

Cell Ratio (Caco-2:HT29)	PMA-COOH		PMA-NH_2	
	Pre	Post	Pre	Post
1:9	0.79 ± 0.06	0.77 ± 0.03	0.74 ± 0.05	0.94 ± 0.09
5:5	0.87 ± 0.05	0.69 ± 0.52	0.81 ± 0.06	0.91 ± 0.09
9:1	0.7 ± 0.09	0.55 ± 0.36	0.72 ± 0.06	1.15 ± 0.14*

[1]Mean ± SD of at least four inserts, *different than COOH (P<0.05).

15. Table S6. Nanoparticle translocation shown as the percent (%) of the amount translocated into the basolateral compartment over a 72-h incubation, relative to the dose administered into the apical compartment.[1]

Cell Ratio (Caco-2:HT-9)	PMA-COOH	PMA-NH_2
1:9	1.37 ± 0.459	3.08 ± 0.168*
5:5	1.29 ± 0.237	2.71 ± 0.683*
9:1	0.080 ± 0.063	3.06 ± 1.26*

[1]Mean ± SD of at least four inserts, *different than COOH in the same cell ratio configuration (P<0.05).
